# Single-Cell Multi-Modal Differential Analysis of the Human Neo-Cortex in HIV Infection Reveals Similarities with Hallmarks of Alzheimer’s Disease

**DOI:** 10.21203/rs.3.rs-7636879/v1

**Published:** 2025-11-01

**Authors:** Arpita Joshi, Pietro Paolo sanna

**Affiliations:** The Scripps Research Institute; The Scripps Research Insititute

## Abstract

Cognitive impairment in people with HIV (PWH) remains prevalent despite viral suppression. To provide insights into the cellular mechanisms of pathogenesis, we carried out a multi-modal pan cell-type specific differential analysis of the frontal cortex of PWH. We show cell type-specific dysregulations of oxidative phosphorylation, glycolysis, ribosomes and translation, DNA damage, and neuroinflammation in PWH. Key genes and pathways identified showed a considerable overlap with transcriptional hallmarks of Alzheimer’s disease (AD) and involved AD vulnerable cell types, among others. We computed several differentially accessible chromatin sites in all major cell-types. Neuronal genes with perturbed chromatin accessibility regions were enriched in synaptic signaling genes supporting an epigenetic contribution to cognitive impairment in HIV. Convergent mechanisms of pathogenesis between HIV and AD support that broad therapeutic targets can be identified to ameliorate neurodegeneration and neuroinflammation in HIV and neurodegenerative conditions such as AD.

## INTRODUCTION

HIV remains a major public health concern^1^. Antiretroviral therapy (ART) has been effective in suppressing HIV replication, particularly in the periphery^2^. ART substantially reduced HIV-related morbidity and mortality and transformed HIV into a manageable chronic disease^3^. While declining, neurocognitive impairment (NCI) in PWH on ART remains prevalent^4^. Prior bulk RNA-seq studies on HIV-infected patients revealed key gene expression changes in the central nervous system (CNS) including immune activation, particularly IFN signaling, as a major factor in HIV’s neurological effects, even without clinical neurocognitive deficits and altered synaptic transmission and neuronal function pathways^5–8^. Other studies linked brain HIV RNA load to downregulation of oxidative phosphorylation, electron transfer, and the tricarboxylic acid (TCA) cycle in PWH^6,9,10^. The causes of NCI in the setting of viral suppression remain elusive and likely include low-level viral replication in the CNS, neuroinflammation, ART neurotoxicity, and prior neurological damage^2,11,12^. Genome-wide strategies still remain underutilized in the neuroHIV field^13^. Thus, there is a pressing need for a better understanding of the pathogenesis of NCI in the current era that can inform new and more effective strategies to improve neuropsychological functioning in PWH.

In this study, we leverage snRNA-seq and scATAC-seq from the Single Cell Opioid Responses in the Context of HIV (SCORCH) consortium^13^ to perform differential analysis of gene expression and chromatin accessibility sites across cell-types from PWH in comparison with uninfected controls. In both excitatory and inhibitory neurons, we found several genes and pathways indicative of neuronal damage and neurodegeneration, including dysregulation of ribosomal proteins and translation, pathways related to neurodegeneration, including AD, Huntington’s disease (HD), Parkinson’s disease (PD) and prion disease. We observed transcriptional evidence of impaired energy metabolism across cell classes including downregulation of glycolysis, oxidative phosphorylation and electron transfer. Glycolysis was downregulated in astrocytes and oligodendrocytes that produce excess lactate to support neuronal energy demands via the astrocyte-neuron lactate shuttle (ANLS), and in selected neuronal subtypes such as the high-frequency firing Pvalb interneurons. Several upregulated pathways are indicative of inflammation and responses to cytokines, interferons and the complement system. We identified several molecular pathways that are altered in specific cell-types, including pathways related to inflammatory responses in immune and glial cells and neurodegeneration and neuroinflammation in neurons, providing insights into the cellular drivers of neuroHIV pathogenesis and potential therapeutic targets.

## MATERIALS AND METHODS

### scVI and Extensions for Differential Analysis

To analyze the snRNA-seq data used in this work, we used the powerful scVI (single cell variational inference)^14,15^ tool, version 1.0.4. It is built on PyTorch^16^ and AnnData^17^ frameworks that use hierarchical Bayesian modeling^18^ to transform the cell-by-gene data into a latent space and facilitate a number of downstream analysis tasks like clustering, visualization, differential expression, etc. The model itself is essentially a variational autoencoder^19^ that models the expression values for each cell across genes using a ZINB (zero-inflated negative binomial) distribution. Quality control to filter the noisy cells was performed first for a threshold for the number of genes with non-zero raw counts and the doublets were removed by training the SOLO model^20^ which along with a variational auto-encoder, employs an additional last neural network layer to feed forward the learned embeddings from the auto-encoder to build a classifier to predict the doublets in the data. The genes were filtered based on whether they were protein coding or not using GENCODE^21^ reference annotation, version 44. Finally, we trained the scVI model with default hyperparameters to compute DEGs (differentially expressed genes). To analyze the ATAC-seq data, we used an extension of the scVI model, the PoissonVI model^22^ with default hyperparameters that uses fragment counts to compute differential accessibility using a similar variational autoencoder architecture as the scVI model that produces differentially accessible regions (DARs) for chromatin accessibility among PWH.

### Other Bioinformatics Tools and Methods

We used Python version 3.11.4 and R version 4.3.0. To find the significance of differentially expressed genes, we used Python’s implementation of Gene Set Enrichment Analysis (GSEA)^23^, gseapy, version 1.0.6. We used the following collections for pathway analysis: Gene Ontology (Biological Process 2023 (GO: BP), Cellular Component 2023 (GO:CC), Molecular Function 2023 (GO: MF), 'WikiPathway_2023_Human' 'MSigDB_Hallmark_2020', 'KEGG_2021_Human'. The pathways delineated in GSEA results are filtered at Nominal p-value 0.01. Consequently, we observed the network perturbations of these transcription factors in the transcriptional signature of each AD stage. We also used Metascape^24^ to further validate pathway analysis results and identify transcription factors from the TRRUST^25^ database for immune and neuronal cells. To evaluate neuronal DEGs with DARs, we used the SynGO^26^ web tool to test the enrichment of synaptic genes.

### Subjects

We analyzed single-nucleus RNA-sequencing (snRNA-seq) samples from the SCORCH consortium, which aims to understand the potentially intertwining biological mechanisms of substance use disorder (SUD) and HIV^13^. Our cohort consisted of pre-frontal cortex area BA-9 (Brodmann area 9) samples from 26 individuals (13 PWH and 13 control samples) who were virally suppressed with plasma HIV RNA levels < 20 copies/mL for >1 year^27^. Both the PWH and the control group had more male samples; more details regarding CD4 counts, ART, cognitive status, etc. of PWH in this work can be found in Supplementary Table-1.

## RESULTS

### Transcriptional Landscape of snRNA-seq Data

**Figure-1:**
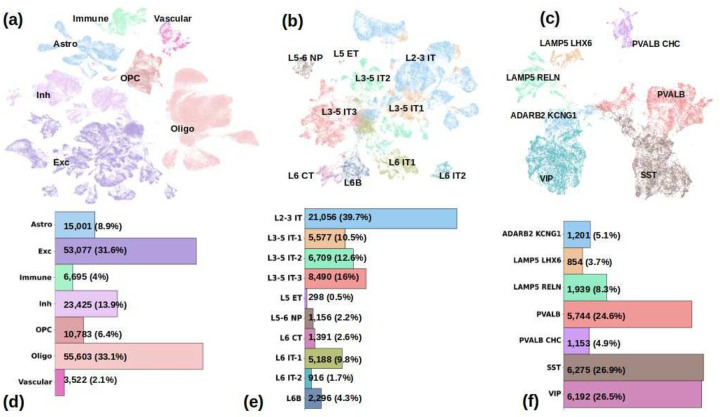
Summary of single-cell RNA-seq data within 26 samples from PWH and controls. (a) Joint UMAP of 168,106 cells across 7 major cell classes including excitatory neurons (Exc) including L2–3 IT, L3–5 IT1, L3–5 IT2, L3–5 IT3, L5 ET, L5–6 NP, L6 CT, L6B, L6 IT1 and L6 IT2; inhibitory neurons (Inh) including Lamp5 Lhx6, Lamp5 Reln, ADARB KCNG1, Vip, Sst, Pvalb and Pvalb Chc; oligodendrocytes (Oli); oligodendrocyte precursor cells (OPC); astrocytes (Astro); immune-related cell types (Immune) including microglia, macrophages, T-cells, B-cells and myeloid cells; vascular and epithelial cells (Vascular) including endothelial, pericytes, smooth muscle cells and vascular leptomeningeal cells; (b, c) UMAP of excitatory (b) and inhibitory (c) neurons; (d-f) Bar plot showing the number of cells per major cell class (d), excitatory neuron cell types (e) and inhibitory neuron cell types (f).

We used the raw UMI (unique molecular identifiers) counts data of the 26 samples (including 13 samples of PWH) from the SCORCH consortium^13^. After filtering out low quality cells and removing doublets using SOLO^20^, a total of 168,106 cells remained, Figure-1 (a) shows the UMAP embedding of the entire dataset and Figure-1 (d) shows their cell-type composition. Based on marker gene analysis, Leiden clustering and previously published data^28,29^, 30 cell-types were annotated in 7 major groups. These included 10 excitatory (Exc) neuron cell types and sub-types, namely, L2–3 IT (layer 2–3 intra-telencephalic), L3–5 IT1, L3–5 IT2, L3–5 IT3, L5 ET (extra-telencephalic projecting also known as L5 pyramidal tract), L5–6 NP (near projecting), L6 CT (corticothalamic), L6B, L6 IT1 and L6 IT2 (53,077 cells, 31.6% of total, Figure-1 (b) and (e) for more details); 7 inhibitory neuron cell types and sub-types, namely, Lamp5 Lhx6 (lysosomal associated membrane protein), Lamp5 Reln, ADARB KCNG1, Vip (vasoactive intestinal polypeptide), Sst (somatostatin), Pvalb (parvalbumin) and Pvalb Chc (Inh, 23,425 cells, 13.9% of total, see Figure-1 (c) and (f) for more details); oligodendrocytes (55,603 cells, 33.1% of total); oligodendrocyte precursor cells (OPC, 10,783 cells, 6.4% of total); astrocytes (15,001 cells, 8.9% of total); 5 immune-related cell-types including microglia, macrophages, T-cells, B-cells and other myeloid cells (6,695 cells, 4% of total); and several vascular cell-types (3,522 cells, 2.1% of total) including endothelial, pericytes, smooth muscle cells and vascular leptomeningeal cells.

### Differential Analysis of Gene Expression

**Figure-2:**
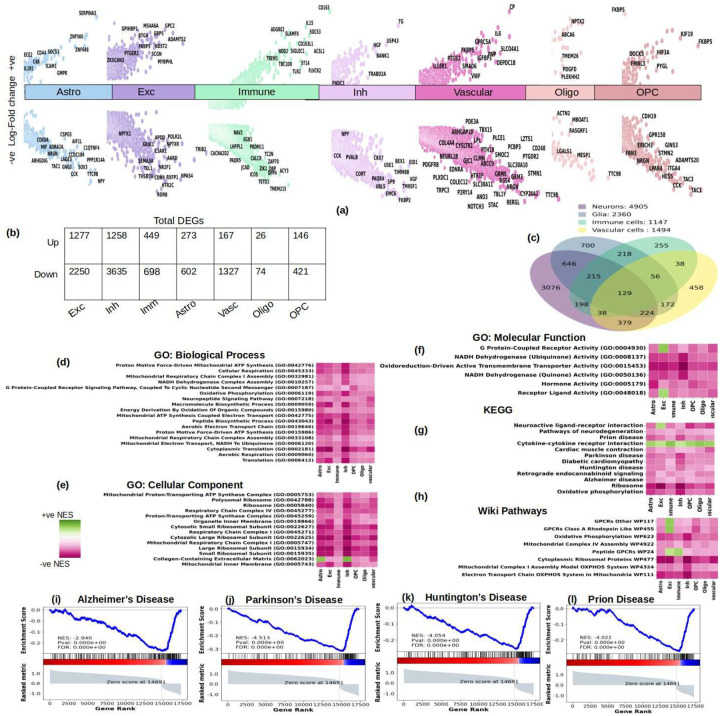
Summary of differential analysis of gene expression data in seven major cell-type families, highlighting the neurodegenerative impact of HIV even in virally suppressed individuals. (a) Genes with maximum significant changes in the seven cell-type families. (b) Total number of significant (filtered at p-value 0.05) DEGs (differentially expressed genes) found in each cell-type family. A predominant downregulation was observed, particularly in vascular cells and neurons, suggesting a broad inhibitory effect on gene expression. (c) Intersection of the overall set of genes in the neurons, glia, immune cells (microglia, macrophages, T-cells, B-cells and myeloid cells) and vascular cells. Neurons had the maximum number of genes unique to them while glia had a relatively smaller percentage of genes unique to them (700 out of 2360). (d, e, f, g, h) GSEA pathways that are commonly dysregulated in all cell-type classes (FDR <= 0.05). (d) Gene Ontology Biological Processes; (e) Gene Ontology Cellular Component; (f) Gene Ontology Molecular Function; (g) Kyoto Encyclopedia for Genes and Genomes; (h) Wiki Pathways; (i, j, k, l) GSEA plots indicative of the downregulation of pathways of neurodegeneration in excitatory neurons, the GSEA algorithm was employed using the differential expression signature of all the excitatory neurons’ subtypes and for genes dysregulated in multiple subtypes we retained the signature indicative of maximum dysregulation - (i) Alzheimer’s Disease, (j) Parkinson’s Disease, (k) Huntington’s Disease, and (l) Prion Disease. The leading edge genes that drive the pathways in (i) through (l) are shown in Supplementary Figure-1.

We compared gene expression levels in PWH versus Control samples by cell-type and identified 6,802 unique differentially expressed genes (DEGs) that implicated all major cell types, see Figure-2 for a summary of the most significant results, the genes curated in Figure-2 (a) have been confirmed for expression in the respective cell-type in The Human Protein Atlas^30^. Most cell-types showed a strong signature of repression— 64% of DEGs in excitatory and 74% in inhibitory neurons were downregulated, 89% of DEGs in vascular cells, 61% of DEGs in immune cells (comprising microglia, macrophages, T-cells, B-cells and myeloid cells). The numbers of DEGs in glial populations were substantially smaller, yet 69% DEGs in astrocytes and 74% DEGs in oligodendrocytes are downregulated. A significant proportion of DEGs (72%) were perturbed in either neurons or glial cells, indicating that changes in gene expression in HIV are mostly cell-type specific.

In astrocytes we found that *SERPINA3* (alpha-1-antichymotrypsin, α1ACT) was the most upregulated gene in PWH (Figure-2). *SERPINA3* was previously unrecognized in neuroHIV, but is upregulated in early and intermediate AD^31^ and is considered to be a marker of neuroinflammation in AD^32^. In AD patients and animal models, *SERPINA3* expression was found to be inversely correlated with the expression of synaptic markers^33^. *SERPINA3* is also recognized as an astrocyte factor contributing to blood brain barrier (BBB) disruption^34^. In astrocytes, we also found upregulation of *IL1R1* and *SOCS3* (Suppressor of Cytokine Signaling 3), both of which are increased in AD brains^35–37^. We also found elevated *ECE2* and *CD44* in astrocytes, *ECE2* (endothelin-converting enzyme-2) is increased in AD brains^38^ and astrocytic *CD44* is a modulator of neuronal excitability in epilepsy^39^. Amongst the downregulated genes in astrocytes, we also found the synapse-associated proteoglycan *CSPG5* (neuroglycan C), deficiency of which is associated with impaired presynaptic function and elimination of synapses^40^. In astrocytes of PWH, we also found downregulation of *AIF1L*, a gene previously shown to be downregulated in astrocytes by inflammatory mediators^41^.

Among the genes induced in excitatory neurons of PWH were *ADAMTS2*, whose over-expression predicts cognitive decline in AD^42^ and *GPC2* that is upregulated in AD^43^. Among the genes downregulated in excitatory neurons we found the serotonin (5-HT)_2C_ receptor (*HTR2C)*, which is involved in synaptic plasticity and memory, impulse control, cognitive flexibility, and attentional processes^44^ and is considered to be a therapeutic target for depressive and anxious states^45^ and AD^46^. Also downregulated in excitatory neurons was *RXFP1*, which is reduced in the cortex of non-depressed AD patients^47^. Other genes downregulated in excitatory neurons of PWH and implicated in neuronal vulnerability in AD include *RORB*, *CDH9*^48^, *CRH*^49^, and *NPAS4*^50,51^.

In inhibitory neurons, we found downregulation of several genes that are also recognized markers of neurodegeneration in AD and other conditions like *SST, PVALB, VGF, CRH*, and *NPY*. Somatostatin (*SST*) was downregulated in inhibitory neurons in the cortex of PWH and *SST* interneurons are vulnerable to degeneration in AD^52,53^. Parvalbumin (*PVALB*) is a calcium binding protein primarily expressed in high-frequency firing inhibitory neurons, which are also vulnerable in AD and other neurological diseases such as many psychiatric disorders, such as schizophrenia, autism spectrum disorders, and substance abuse^52,54–56^. Also downregulated in inhibitory neurons of PWH is the neurotrophin-induced protein *VGF*, which is involved in regulating energy balance, neuroplasticity and neuroprotection and is also downregulated in AD and other neurodegenerative diseases^57,58^. *NPY* and *CRH*, previously recognized for their association with neurological damage and AD, were downregulated in inhibitory neurons as well^31,49,59^. We also found cortistatin (*CORT*), a peptide structurally similar to *SST*, to be downregulated in inhibitory neurons of PWH. *CORT* has been found to reduce neuroinflammation and BBB disruption^60^. We found downregulation in PWH of another interneuron marker gene, cholecystokinin (*CCK*), which is downregulated in AD with cognitive impairment^61^. Among the upregulated genes in inhibitory neurons, we found *HGF*, which has been found to be upregulated in AD patients with mild cognitive impairment^62^ and it is protective from neuronal apoptosis^63^.

Recognized markers of neuroinflammation were also found to be upregulated including *SOCS3* and *IL-15* in immune cells^37^, and *IL-6* in vascular cells^64^. *NPTX2*, which was downregulated in excitatory neurons but upregulated in oligodendrocytes, has been shown to be downregulated in excitatory neurons in advanced AD^29,31^ and upregulated in non-neuronal cells in early AD^31^.

While differential gene expression in PWH was generally cell-type specific, we found that some of the top DEGs are involved in related processes across cell types. We found pathways indicative of neurodegeneration, like AD, Parkinson’s Disease (PD), Huntington’s Disease (HD), prion disease, Amyotrophic Lateral Sclerosis (ALS), and pathways indicative of neuro-inflammation like, cytokine-receptor activity and interferon signaling, and oxidative phosphorylation dysregulated in all seven cell-type families, see Figure-2 (d) through (h). Among broadly dysregulated pathways across cell types were also pathways related to ribosomes and translation, Figure-2 (d, e, g). Dysfunctional ribosomal biogenesis has been implicated in aging and neurodegenerative diseases^65^. A complete list of pathways significantly perturbed (nominal p-value <= 0.01) in all the cell-types can be found in Supplementary Tables 2 through 6.

**Figure-3:**
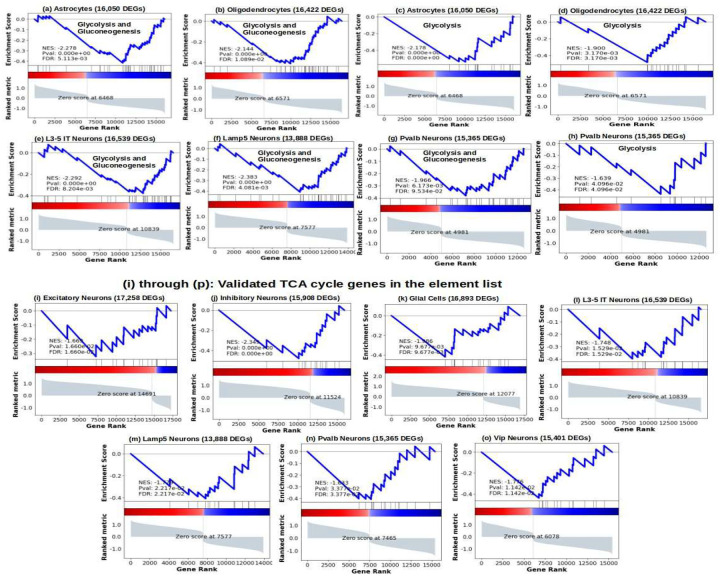
Summary of energy metabolism in neuronal and glial cells. Each enrichment test has the number of DEGs in parentheses in the full list, the normalized enrichment score is negative for all comparisons. (a, b, e, f, g): enrichment plots in glia and neurons of the WikiPathway for Glycolysis and Gluconeogenesis in (a) Astrocytes, (b) Oligodendrocytes, (e) L3–5 IT neurons, (f) Lamp5 neurons, and (g) Pvalb neurons. (c, d, h): enrichment plots in glia and neurons of the curated Glycolysis only genes in, (c) Astrocytes, (d) Oligodendrocytes, and (h) Pvalb neurons. (i) through (o): enrichment plots of TCA cycle genes (p-value <= 0.05), (i) Excitatory neurons, (j) Inhibitory neurons, (k) Glia cells, (l) L3/5-IT neurons, (m) Lamp5 neurons, (n) Pvalb neurons, (o) Vip neurons. The maximally significant enrichment of the TCA cycle gene-set was seen in the inhibitory neurons, when considered altogether, followed by glial cells when considered altogether.

We also found electron transport and oxidative phosphorylation to be significantly downregulated across cell-type families, Figure-2 (g). Energy metabolism has been shown to be majorly impacted in PWH^6,9,10^. To further validate the overarching presence of oxidative phosphorylation, electron transfer and other energy metabolism related pathways, in all cell-type families, we studied various gene-sets of energy metabolism in the differential expression signature of all the cell-types. We show, in Figure-3 (a) through (h), the WikiPathway gene-set representative of glycolysis and gluconeogenesis and another gene-set composed of glycolysis-only genes (*ALDOA, BPGM, ENO1, ENO2, GAPDH, GPI, HK1, HK2, HKDC1, PFKL, PFKM, PGAM1, PGAM2, PGAM4, PGK1, PKLR, PKM, TPI1*) were significantly negatively enriched in astrocytes and oligodendrocytes and a few neuronal populations including the fast-firing Parvalbumin interneurons. Furthermore, we found genes involved in TCA cycle, most of which were shown to be downregulated in the frontal cortices of PWH with a high HIV mRNA load^6^, namely, *ACO2, IDH3A, IDH3G, OGDH, SUCLA2, SDHB, FH, MDH1, MDH2, GAPDH, ENO1, SDHA, ATP5G3, GLS, ACO1, PDH1, CS*, to also be negatively enriched in multiple neuronal and glial cell-types, in Figure-3 (i) through (o). In particular, glycolysis gene-sets were significantly downregulated in astrocytes and oligodendrocytes that produce excess lactate to support neuronal energy demands (ANLS), which indicates impaired neuron-glia metabolic cooperation that is implicated in impaired neuronal plasticity and memory and several neurological diseases^66^. Glycolysis gene-sets were also downregulated in selected neuronal subtypes, like L3/5 IT excitatory neurons, and interneurons like Lamp5 and the high-frequency firing Pvalb, Figure-3 (a) through (h). Likewise, TCA cycle gene-set was downregulated in glial cells and various neurons including L3/5 IT, Pvalb, Vip, and Lamp5. TCA cycle downregulation is also observed in AD^67^. In addition to disrupting production of reducing equivalents for electron transfer and oxidative phosphorylation^6,9^, it has been shown to influence chromatin modifications, DNA methylation and protein post-translational modifications^68^.

### Immune Cells Response to HIV Infection

**Figure-4:**
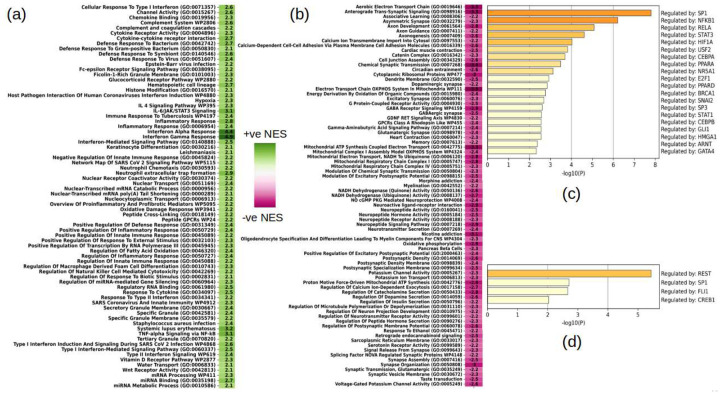
Summary of pathway analysis in immune-related cells and enrichment of transcription factor targets in the TRRUST^25^ database dysregulated in immune cells. (a, b) Selected statistically significant (filtered at FDR corrected p-value of 0.01) up (a) and down (b) regulated gene sets; (c, d) Enrichment of transcription factor targets in the significantly up (c) and down (d) regulated DEGs in immune cells.

To increase the power of the differential analysis (due to fewer number of individual cells of each category) and have a holistic view of the immune responses in the context of HIV, we analyzed in aggregate the cells known to perform immunological functions in the central nervous system, namely, microglia, macrophages, other myeloid cells, T-cells, and B-cells. Combined differential analysis of these cells revealed a significant upregulation of several inflammation related pathways and defense responses to viruses and bacteria like cytokine receptor activity, type I and type II interferon signaling, IL-4/IL-6 signaling, TNF-a, among others, see Figure-4 (a). Likewise, complement system which has been implicated in neuroinflammation, neurodegeneration as well as neuroprotection and infectivity^69,70^. The vitamin D receptor pathway was upregulated in immune cells, while it was downregulated in excitatory neurons (see next section). Hypovitaminosis D is prevalent in people living with HIV-1 and has been linked to HIV progression^71^. Also significantly downregulated were pathways related to oxidative phosphorylation, electron transport, ATP synthesis, NADH activity (Figure-4 (b)). A list of all the significantly perturbed (nominal p-value <=0.01) pathways in immune cells can be found in Supplementary Table-6.

We next used the TRRUST^25^ database to identify transcription factors governing the DEGs identified, Figure-4 (c) and (d) show our findings in up and down regulated DEGs in immune cells, respectively. The top results include *SP1, NFKB1, REST, STAT3, PPARA*, and *GATA4*, among others. *SP1* is known to modulate transcription in response to physiological and pathological stimuli, it also binds with high affinity to GC-rich motifs and regulates the expression of many genes involved in a variety of processes such as cell growth, apoptosis, differentiation and immune responses and is also highly regulated by post-translational modifications^72–74^. *NFKB1* is the final response to several signal transductions particularly in inflammation, immunity, differentiation, cell growth, tumorigenesis and apoptosis^75,76^; NF-kB signaling is also involved in HIV expression^77^, and has been found to correlate with HIV RNA load in the brain of people with HIV^6^ and is dysregulated in advanced AD^31^. *REST* is a transcriptional repressor that binds to the neuron-restrictive silencer (NSR) element and inhibits the transcription of neuronal genes in non-neuronal cells^78–82^. Lastly, *STAT3* signaling, Figure-4 (a), is a major mediator of inflammation that is activated by NF-κB target genes, such as IL-6 and contributes to T-cell malignancies^6,83–85^.

### Neuronal Responses to HIV Infection

**Figure-5:**
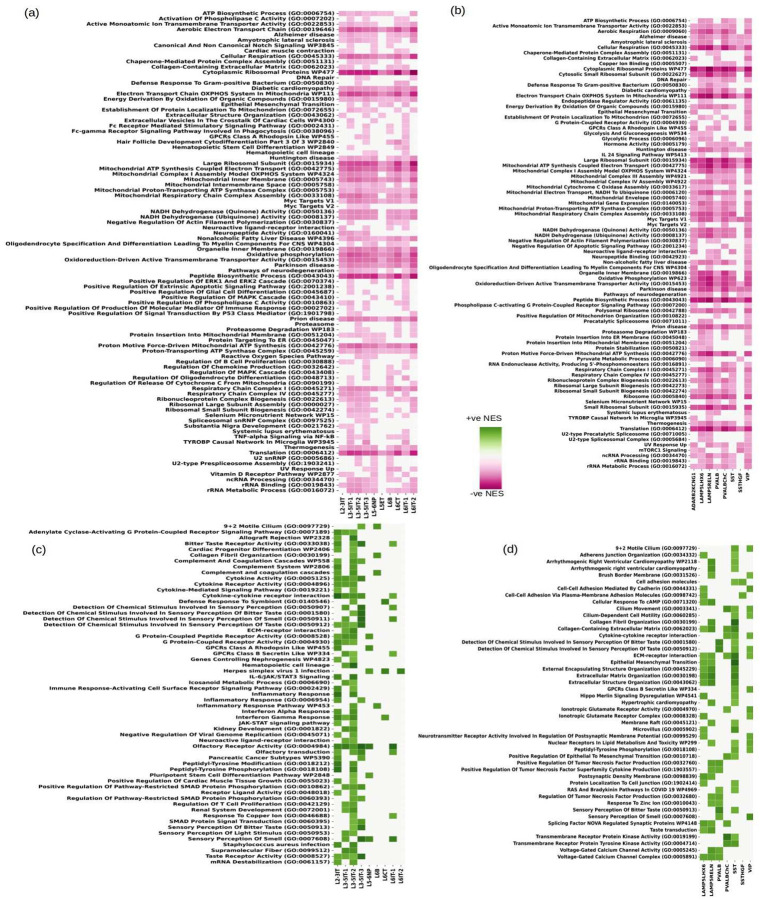
Selected GSEA pathways dysregulated in excitatory and inhibitory neurons. (a, b) Down regulated consensus GSEA pathways that are statistically significant in excitatory neurons (a) and inhibitory neurons (b). (c, d) Up regulated consensus GSEA pathways that are statistically significant in excitatory neurons (c) and inhibitory neurons (d). The consensus pathways are the ones that are significantly perturbed in multiple excitatory (or inhibitory) neuron sub-cell-types.

In both excitatory and inhibitory neurons, we found several pathways indicative of neuronal damage and neurodegeneration, including dysregulation of cytoplasmic ribosomal proteins and translation, pathways related to neurodegeneration, including AD, HD and PD, inflammation and impaired energy metabolism including downregulation of oxidative phosphorylation, electron transfer, that are seen across neuronal cell types. The results are summarized in Figure-5, where we show up and down regulated consensus pathways, which for both the neuronal classes refer to the ones that are perturbed in multiple cell-types. Several upregulated pathways, shown in Figure-5 (a, b) are indicative of neuronal responses to cytokines, interferons, and the complement system. We also found significant dysregulation of pathways indicative of chemo-sensing dysfunction, including, chemical stimuli affecting sensory perception of bitter smell and taste, olfactory receptor activity, taste/olfactory transduction, etc., Figure-5 (c, d). Several of the olfactory receptor genes that drive the chemo-sensing pathways downregulation are shown in Supplementary Figure-2. In excitatory neurons, we found vitamin D receptor pathway downregulated, Figure-5 (a), and vitamin D deficiency has been linked to AD and other neurodegenerative diseases^86^. Similarly, MYC targets V1 and V2 were also downregulated in both the neuronal classes and have been shown to be downregulated in AD as well^87^. Inhibitory neurons also exhibit dysregulation of pathways related to heavy metal absorption, like zinc and selenium (Figure-5 (b) and Supplementary Table-3), which can exacerbate cognitive decline^88 89^. A complete list of significantly perturbed (nominal p-value <=0.01) pathways in excitatory and inhibitory neurons can be found in Supplementary Table-2 and Supplementary Table-3 respectively.

**Figure-6:**
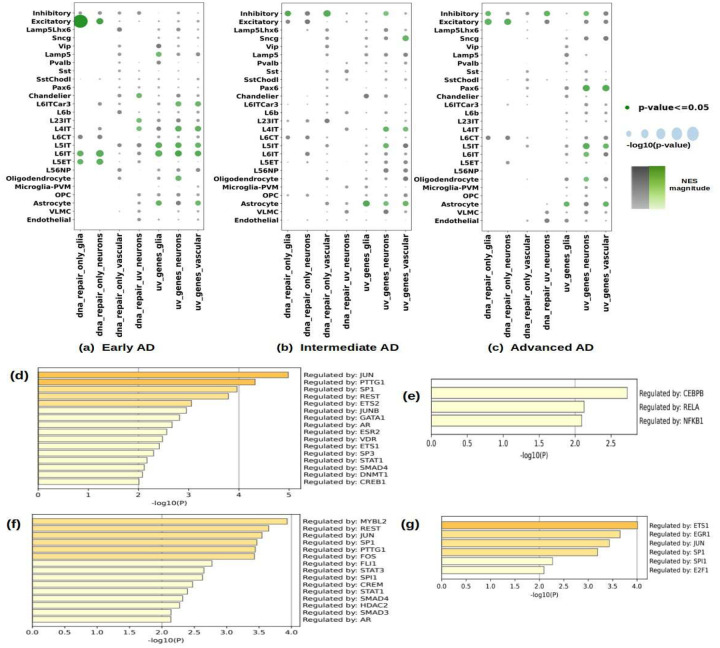
Enrichment analysis of curated gene-sets and transcription factor targets. (a, b, c) Along X-axis, we have gene-sets procured from differential analysis of HIV data, in the distribution of DEGs in the corresponding cell-types in the three AD stages from^31^. The enrichment was performed for the 24 cell-types in the AD data and the two neuronal classes taking all the excitatory and inhibitory subclasses together. Except for the manually curated ’dna_repair_uv_neurons’ gene-set, all the rest are pre-defined in the databases used for the pathway analysis mentioned in the Methods. (d, e, f, g) Enrichment of transcription factor targets in the TRRUST^25^ database dysregulated in the two neuron classes. (d, e) Enrichment of transcription factor targets in the significantly down (d) and up (e) regulated DEGs in excitatory neurons; (f, g) Enrichment of transcription factor targets in the significantly down (f) and up (g) regulated DEGs in inhibitory neurons.

Another significant finding in both the neuronal classes was the down regulation of the UV response pathway, see Figure-5 (a) and Figure-5 (b), which include a number of genes that are known to contribute to DNA damage namely, *POLR2H, EPHX1, BID, SQSTM1, EPCAM, ATP6V1C1, CHRNA5, GGH, TUBA4A, DNAJB1, H2AX, RET, POLE3, BTG1, BTG2, BTG3, SOD2, FEN1, IGFBP2, ABCB1, GPX3, BMP2, CDO1, PARP2, NR4A1, RFC4, LYN, FOS, ALDOA, IRF1, BSG, POLG2, CDC34, HSPA2, CDC5L, AGO2, DDX2*^90–125^. We tested this gene-set termed ‘dna_repair_uv_neurons’ in Figure-6 (a) through (c). Other DNA repair gene-sets are also significantly downregulated in both inhibitory and excitatory neuronal classes, Figure-5 (a, b). Since dysfunctional DNA repair contributes to the pathogenesis of neurodegeneration including AD^126,127^, we curated gene-sets from various cell-type families consisting of genes that are the leading-edge genes of UV response pathways and DNA repair pathways and conducted an enrichment analysis in the differential expression signature of three clinically defined AD stages as from the results of^31^. The present gene-sets of these pathways from various cell-type families are not mutually exclusive but have a small union set. The enrichment analysis was performed using the GSEA algorithm, which performs the Kolmogorov-Smirnov test to evaluate whether the gene set is drawn from the distribution of DEGs in the corresponding AD stage. The results are summarized in Figure-6 (a) through (c), which shows that maximum negative enrichment of these gene-sets is found in early AD. We performed the test for individual cell-types in the AD dataset and also evaluated the results for all excitatory and inhibitory neurons’ subtypes altogether; the excitatory neurons in early AD in the DNA repair gene-set from glial cells in HIV had the maximally significant enrichment, they also had significant enrichment for DNA repair gene-set of neurons. Intra-telencephalic neurons in early AD, L4 IT, L5 IT, L6 IT, L6 IT Car3 had significant enrichment of UV response genes from neurons, glia and vascular cells as well. Relatively, intermediate and advanced AD had enrichment of these gene-sets in fewer cell-types, with DNA repair gene-sets finding significance only if excitatory and inhibitory neurons are considered together and UV gene sets find significance in some individual cell-types, like Sncg, L4 IT and L5 IT in intermediate AD, and Pax6, L5 IT and L6 IT in advanced AD. Interestingly, in all three AD stages, astrocytes had significant enrichment for glial and vascular UV response gene-sets from HIV. The subset of neuronal UV response gene-set, the ‘dna_repair_uv_neurons’, its enrichment indicates that there are genes in UV response pathways that are not in DNA Repair pathways indicative of a specific molecular mechanism of impaired DNA repair in PWH that is concordant to changes in various AD stages.

We then queried the TRRUST database, like we did for immune cells, to identify transcription factors governing the DEGs identified in excitatory and inhibitory neurons, Figure-6 (d) through (g). We found overlap with the transcription factors identified in immune cells, including *SP1, REST, STAT1, GATA*, among others. We found the transcription factors *JUN* and *FOS* that are known to work in tandem and can cause neuronal apoptosis in AD^128^. *JUN* also interferes with axon regeneration^129^ and both participate in transcriptional regulation that plays a key role in neuronal responses to external stimuli^130^ that include protein phosphorylation- a leading AD marker^131^. *JUN* has also been found to interact with HIV Tat^132,133^. We also found SMAD proteins, *SMAD3* and *SMAD4*, that are involved in neuroinflammation and neuronal apoptosis^134–136^ and also regulate transcriptional effects of transforming growth factor β, which in turn is activated by HIV-1 Tat protein^137^, and we also found transcriptional evidence of increased SMAD protein phosphorylation in L3–5 IT and somatostatin neurons, see Figure-5 (c) and Supplementary Table-3 respectively. Maximally significant transcription factor governing DEGs in inhibitory neurons was *MYBL2*, which has been implicated in promotion of neuronal apoptosis^138–140^ and in immune cells it has been found dysregulated in myeloid cell division and malignancies^141^ and has been found to be associated with multiple intracellular organelle and cell-death related pathways in a differential gene analysis of CD3+ T-cells in HIV patients with varied levels of disease progression^142^. We also found *ETS1* and *ETS2*, respectively, in excitatory and inhibitory neurons known to be associated with neuronal differentiation^143^ and in T-cells they have been shown to promote HIV replication^144^. Likewise, we found *PTTG1* as a regulator of downregulated target genes of both the neuronal classes, which has been found to be dysregulated in a model of stroke and fetal alcohol syndrome^145,146^.

### Glial Responses to HIV Infection

**Figure-7:**
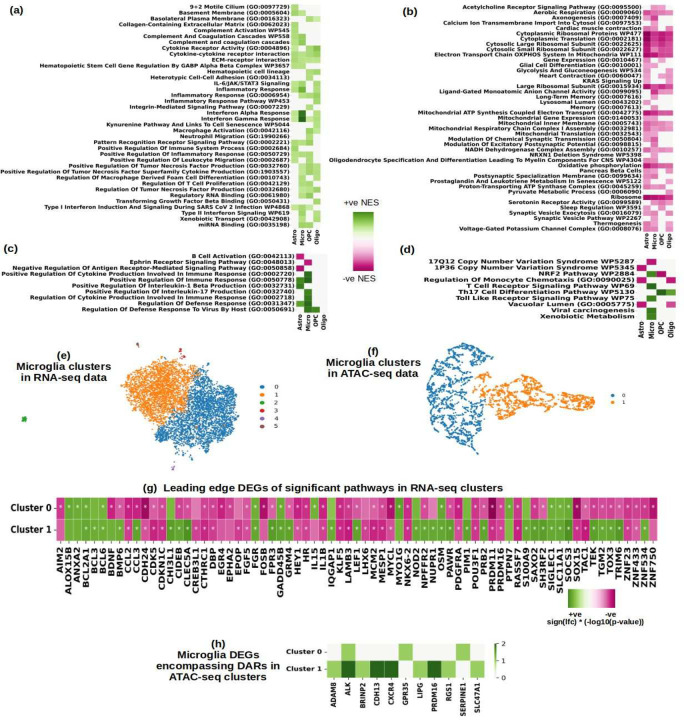
Summary of glial dysregulation in HIV. (a, b) Selected statistically significant (filtered at FDR corrected p-value of 0.05) up (a) and down (b) regulated GSEA pathways in glial cells; (c, d) Selected differentially regulated pathways in glial cells that are exclusive to glial cell-types. (e, f) Two significant microglial clusters in RNA-seq (e) data and ATAC-seq (f) data. (g) DEGs significantly dysregulated in at least one of the two RNA-seq clusters that also drive the most significantly perturbed pathways in the two RNA-seq clusters, * denotes that the gene is significantly differentially expressed in the corresponding cluster. ‘Cluster 0’ in RNA-seq data has more inflammatory pathways and ‘Cluster 1’ has more cell-death related pathways. (h) Genes that encompass at least one DAR in one of the two ATAC-seq clusters.

The enriched pathways in glia are also resonant of general changes in other cell-type families, Figure-7 (a, b). These changes include reduction in mitochondrial respiration, increased inflammation, and reduced protein syntheses. Amongst the inflammation related upregulated pathways, the maximum upregulation was seen in microglia, as can be seen in Figure-7 (a). These pathways include interferon type I and II and cytokine signaling, such as IL-6/JAK/STAT3, and complement. Other noteworthy, upregulated pathways include extra-cellular matrix interactions, integrin mediated signaling^147^, hematopoietic cell lineage^148^. In the downregulated pathways, Figure-7 (b), we found prevalence of energy metabolism and mitochondrial respiration damage including electron transport, oxidative phosphorylation, NADH dehydrogenase; likewise, cytoplasmic ribosomal protein synthesis was also significantly downregulated in all glial cells, consistent with previous observations in macrophages of PWH and at bulk gene expression level^6,149^. Other significantly downregulated pathways include glycolysis and pyruvate metabolism, and pancreatic beta cells, which together with oxidative phosphorylation indicative of dysregulation of energy metabolism in PWH, as discussed above. In Figure-7 (c) and (d) we show pathways that are exclusively dysregulated in glial cells. In microglia, we found some specific transcriptional evidence of inflammation and activation, including pathways indicative of immune activation, cytokine production and responses to viral infection, toll-like receptor signaling, which is key to microglia activation in both HIV and AD and the consequent release of pro-inflammatory cytokines^150,151^. Other inflammatory pathways that are upregulated in microglial cells, include IL-17 production, while IL-1β was induced in astrocytes IL-1β production. Other pathways exclusively dysregulated in microglia were xenobiotic metabolism, viral carcinogenesis, T cell receptor signaling and toll-like receptor signaling^152^ and ephrin signaling pathway, which is implicated in neurodegeneration^153^. A notable finding was the cell-type specific dysregulation of *NRF2* pathway, which was upregulated in microglia but downregulated in OPCs. *NRF2* regulates cellular responses to oxidative stress, it is also dysregulated in AD, and it is a therapeutic target for reducing *NLRP3* activation and neuroinflammation^154^. Other pathways exclusively perturbed in glial cells were regulation of monocyte chemotaxis, Th17 cell differentiation and vacuolar lumen indicating damaged permeability of cells. A complete list of pathways significantly perturbed (nominal p-value <= 0.01) in glial cells can be found in Supplementary Table-4.

We segregated the microglial cells from both RNA-seq and ATAC-seq data and performed Leiden clustering (full details of ATAC-seq data analysis are presented in the next section). In both the modalities we found two significant clusters; the results are summarized in Figure-7 (e) through (h). For the major clusters so identified, we again performed differential analysis among HIV and control samples. Among the two RNA-seq clusters (Figure-7 (e)), we found ‘Cluster 0’ to be enriched in genes that drive more inflammation related pathways and ‘Cluster 1’ to be more enriched in genes that contribute towards more cell-death related pathways. The genes in the leading edge of inflammatory pathways of ‘Cluster 0’ include *ALOX15B, BCL6, FGR, FPR3, IL15, OSM, SIGLEC1, SLC11A1*, and *TAC1*. The genes in the leading edge of cell-death related pathways of ‘Cluster 1’ were *BCL2A1, BCL3, BCL6, BDNF, BIRC3, CIDEB, CLEC5A, GADD45B, GRM4, LEF1, NUPR1, PAWR, PIM1, SH3RF2, SOCS3, TEK, TGM2*, and *TOX3*. In Figure-7 (g), we denote the genes that are in the leading edge of either of the two clusters and are also significantly differentially expressed in at least one of the clusters. Among the two ATAC-seq clusters (Figure-7 (f)), the DARs we found mapped to the genes shown in Figure-7 (h), namely, *ADAM8, ALK, BRINP2, CDH13, CXCR4, GPR35, LIPG, PRDM16, RGS1, SERPINE1*, and *SLC47A1*.

### Cell-type Specific Association of DEGs with Differentially Accessible Chromatin Sites

**Figure-8:**
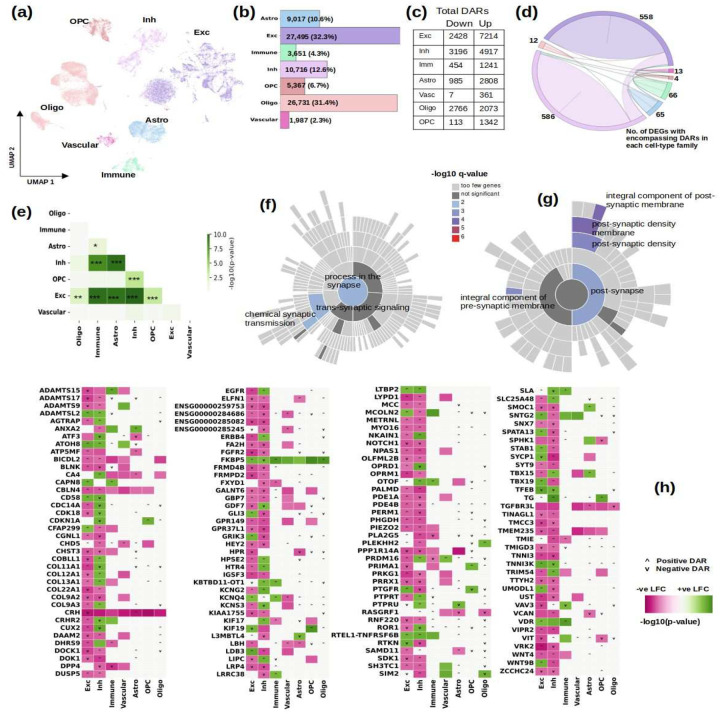
Summary of single-cell ATAC-seq data from 12 PWH and control samples. (a) Joint UMAP of 85,234 cells across 7 major cell classes; (b) Bar plot showing the number of cells per major cell class; (c) Number of differentially accessible regions (DARs) in each cell-type family; (d) Circos plot depicting the number of DEGs containing DARs in each cell-type family, the links represent the number of such DEGs shared between the families, the two neuronal classes share the maximum number of DEGs that encompass DARs within their genomic regions, 111; (e) Fisher exact statistics of DEGs used in part (d) with each cell-type family. The heatmap is drawn with a maximum value of 10 for −log10(p-value) to mask the high p-value of 3.2e-93 between excitatory and inhibitory neurons: *p-value<0.05, **p-value<0.01 and ***p-value<0.001. (f, g) Sunburst plots depicting the enrichment analysis of the neuronal DEGs with DARs using SynGO^26^; (f) the synaptic molecular functions enriched in the list of neuronal DEGs with DARs; (g) the synaptic cellular components enriched in the list of neuronal DEGs with DARs. (h) Cell-type specific DEGs encompassing DARs; for each gene, the color in the heatmap shows the direction of differential regulation. The markers: ’^’ and ’v’ show the direction of differential chromatin accessibility of the maximally significant DAR within the coordinates of the respective DEG. The direction of differential gene expression is not always the same as the direction of differential chromatin accessibility. Some genes might not be significantly differentially expressed in certain cell-types, but the chromatin accessibility of their DARs might still be significant, this state is believed to allow for gene activation in response to environmental cues^166^.

We obtained ATAC-seq based chromatin accessibility data for 6 HIV and 6 control samples from the 26 for which RNA-seq data was available. We obtained a cell-type label at the broad cell-type family level for each cell, corresponding to the RNA-seq data. In all, after quality control, the chromatin accessibility data had 85,234 cells and sequenced 260,424 peaks. For each cell-type family, we filtered out noisy peaks and retained only the top 10,000 highly variable peaks to conduct differential accessibility analysis (see Methods). In a fashion similar to the one adopted for RNA-seq data, we trained a deep learning model to obtain DARs between HIV and control samples (see Methods). Figure-8 shows a summary of cell-types, in Figure-8 (a) we show the UMAP embedding, Figure-8 (b) shows the distribution of their counts, and Figure-8 (c) shows the number of DARs in each directionality for each cell-type. In order to unravel the role of chromatin accessibility in gene expression, we then computed the number of DEGs with at least one DAR within the coordinates of each DEG. The number of such DEGs found in each cell-type along with their intersection is shown in Figure-8 (d, e) and we show the significance obtained using Fisher Exact statistics to evaluate the inter-dependence of these DAR encompassing DEGs among the cell-type families.

We next present a group of genes that stand out from the differential analysis of both gene expression and chromatin accessibility. Figure-8 (h) shows the DEGs with encompassing DARs in more than one cell-type. For genes that harbor multiple DARs within their coordinates, we show the directionality of the DAR that has the maximum significance. There was no significant correlation between the direction of differential dysregulation between gene expression and chromatin accessibility. Some significant findings were: most DEGs with DARs are in neuronal cell-types; downregulation of collagen genes in excitatory neurons and upregulation of some of the same collagen genes in inhibitory neurons; persistent dysregulation of the stress related genes *CRH* (down) and *FKBP5* (up), which likely reflect the increased activation of the hypothalamic–pituitary–adrenal (HPA) that is seen in PWH^155^. Increased HPA activation predisposes to depression and alcohol and drug abuse^156^. HPA activation is also present in AD and other neurodegenerative conditions which have been implicated in disease progression and cognitive decline^157,158^. We also observed neuronal dysregulation of genes involved in voltage-gated potassium channels like *KCNG2, KCNQ4* and *KCNS3*; dysregulation of phosphatase catalytic genes *PTPRT* and *PTPRU*; positive chromatin accessibility and differential expression of T-box transcription factors *TBX15* and *TBX19* in both excitatory and inhibitory neurons; and the neuronal dysregulation of troponin I related genes *TNNI3* and *TNNI3K*.

To further investigate the genes with DARs in neurons, we tested them for enrichment in specific gene sets for synaptic genes from the SynGO database^26^. The results are summarized in the two sunburst plots in Figure-8 (f) and (g) that respectively show the ontology processes and cellular components perturbed by these genes. We found chemical synaptic transmission and components of both pre and post synaptic membranes significantly dysregulated. Synaptic dysfunction has been shown to be prominent in PWH with neurocognitive deficits^6–8^. Some genes that drive these perturbations are shown in Figure-8 (h), *EGFR*^159^, *ELFN1*^160^, *ERBB4*^159,161^, glutamate receptor *GRIK3*, *NOTCH1*^162^, opioid receptors *OPRD1*, *OPRM1*^163,164^ and *PTPRT*^165^. The complete list of synaptic genes with their differential expression in the neuronal subtypes can be seen in Supplementary Figure-3.

## DISCUSSION

We conducted a cell-type specific multimodal differential analysis of the human neo-cortex of PWH. The differential gene expression analysis revealed widespread transcriptional changes across all major cell types, highlighting the extensive molecular impact of HIV infection. Of the 6,802 DEGs identified, the majority displayed repression, particularly in neurons, suggesting a broad inhibitory effect on neural gene expression. We observed reduced expression of gene-sets that are representative of oxidative phosphorylation, electron transfer, ribosomes and translations across all cell types, indicating predominantly catabolic changes. The observed cell-type specific expression changes were particularly pronounced in neurons and glial cells, where maximal DEGs were found, underscoring the vulnerability of these populations to HIV-related pathology. Notably, despite cell-type specificity, many top DEGs were linked to shared pathways, including neurodegeneration-related processes such as AD and PD, and neuroinflammation pathways like cytokine-receptor activity, interferon signaling, and energy metabolism such as oxidative phosphorylation. This supports that the molecular underpinnings of HIV-induced damage may converge on common pathways of neural dysfunction and inflammation across diverse cell types that are at least in part shared with neurodegenerative disorders.

In astrocytes, the upregulation of *SERPINA3*, a marker of neuroinflammation previously unrecognized in neuroHIV but well-documented in AD, is a significant finding. *SERPINA3* contributes to neuroinflammation and blood-brain barrier (BBB) disruption in AD^32^, which suggests a parallel mechanism in HIV, where it may facilitate immune cell activation and infiltration and neuronal damage. The increased expression of inflammatory markers such as IL-1β and *SOCS3*, both implicated in neurodegeneration^36^, further supports the role of astrocytes as key mediators of inflammatory responses in neuroHIV. These findings align with prior studies linking IL-1β to reactive oxygen species (*ROS*) production and *SOCS3* to AD-related neuroinflammation^36^, supporting a shared inflammatory landscape between neuroHIV and neurodegenerative diseases such as AD. We also found transcriptional evidence of increased HPA activation and cortisol secretion in PWH, which predisposes to depression and substance abuse as well as cognitive decline in AD^156–158^. Conversely, the downregulation of genes like *NPY*, *CSPG5, VGF, ADAMTS2* and *GPC2* among others, in astrocytes and other cell-types points to impaired neuroprotective mechanisms and compromised synaptic function contributing to cognitive deficits.

In excitatory and inhibitory neurons, the downregulation of genes such as *HTR2C, RXFP1, PVALB, SST, NPY*, and *CRH*, which are also associated with AD and other neurodegenerative disorders, likely contribute to impaired neuropsychological functioning in PWH and indicates vulnerability of specific neuronal populations in PWH. For instance, downregulation *of HTR2C*, which plays a role in impulse control and cognition^44^, can contribute to executive function deficits observed in HIV associated neurocognitive decline. Similarly, the downregulation of *SST* and *PVALB*, as well as reduced glycolysis in the latter, suggests mechanisms of impaired neural circuit balance and loss of inhibitory control, which are also a hallmark of neurodegeneration in AD^52–55,167^.

In our analysis of immune cells, we combined various immunologically relevant cell types—macrophages, myeloid cells, T-cells, B-cells, and microglial cells—due to the limited number of individual cells per cell-type. Immune cells, particularly microglia, exhibited robust upregulation of inflammatory pathways, including cytokine receptor activity, interferon signaling, TNF-α signaling and the complement system, which is crucial for innate immunity and linked to increased HIV infectivity^70^ and neurodegeneration in PWH^5,6^. Complement activation in the brain contributes to neuroinflammation and excessive synaptic pruning and has emerged as a potential therapeutic target for AD^168^. The activation of these pathways, alongside transcription factors like *SP1, NFKB1, REST*, and *STAT3*, highlights the central role of immune cells in driving neuroinflammation in HIV^6,72–76,83,84^. The upregulation of the vitamin D receptor pathway in immune cells, contrasted with its downregulation in excitatory neurons, is particularly intriguing. Hypovitaminosis D is prevalent in HIV and linked to disease progression, and its differential regulation across cell types may reflect distinct roles in immune activation versus neuronal protection^71,86^. Hypovitaminosis D is also associated with AD disease progression and cognitive impairment in aging^86,169–171^. The downregulation of oxidative phosphorylation and mitochondrial function in microglia can be indicative of a more pro-inflammatory cellular state^172–174^.

The enrichment of pathways related to AD, PD, HD, and prion disease, Figure-2 (i) through (l), across all cell types is a critical finding, along with the downregulation of MYC targets V1 and V2^87,88^ in both excitatory and inhibitory neurons, alongside dysregulated heavy metal absorption pathways (e.g., zinc and selenium) in inhibitory neurons is also seen AD^87–89^. These pathways, including oxidative phosphorylation, ribosomal biogenesis, and DNA repair, suggest that HIV induces a neurodegenerative phenotype even in virally suppressed individuals. Previous studies investigated gene expression patterns associated with neurological disorders in HIV-infected patients at the bulk RNA-seq resolution that demonstrated downregulation of genes related to neurodegeneration was associated with neuroHIV progression^5,6^ as well as the downregulation of genes related to energy metabolism including genes related to oxidative phosphorylation, electron transfer, and the TCA cycle in multiple brain regions^6^. In this work, the significant downregulation of energy metabolism gene-sets (e.g., *ACO2, IDH3A, SDHB*) in neuronal and glial cells, validated against prior studies^6^, supports a role of mitochondrial dysfunction as a central mechanism in neuroHIV in the setting of viral suppressive ART. This is consistent with the observed downregulation of oxidative phosphorylation in immune cells, suggesting a broad metabolic reprogramming of HIV in the neo-cortex. We observed coordinated changes in genes related to bioenergetics in neurons and glia. Downregulation of oxidative phosphorylation genes was observed across both neuronal and glia cell types. Glycolysis genes were primarily downregulated in astrocytes and oligodendrocytes and selected neuronal populations. Astrocytes and oligodendrocytes have high capacity for glycolytic activity that is triggered by neuronal activity^66^. Lactate released by astrocytes in response to neuronal activity is taken up by neurons and used as a supplemental, oxidative substrate, the ANLS^66^. In addition to meeting the energy requirements of active neurons, extracellular lactate released by astrocytes contributes to the regulation of neuronal excitability and plasticity^66^, and thus can contribute to cognitive impairment. Among the few neuronal populations in which glycolysis gene-sets were downregulated are Pvalb inhibitory neurons that are characterized by high-firing frequency that allow them to maintain proper excitation-inhibition balance communication, leading to neuronal damage^134–137^. The significant role of *MYBL2* in inhibitory neurons linked to neuronal apoptosis and cell-division in immune cells, further underscores the complexity of transcriptional regulation in neuroHIV^138–140^. The dysregulation of *ETS1* and *ETS2*, associated with neuronal differentiation and HIV replication, suggests that HIV may hijack developmental pathways to promote pathogenesis^143,144^.

Glial cells, particularly microglia, exhibited pronounced inflammatory responses, with upregulated pathways including interferon signaling, IL-6/JAK/STAT3, and complement system responses, all of which are critical components of the immune response to HIV. Since microglial cells have been studied for their various activation states under HIV^184,185^, we also analyzed them separately. The identification of two microglial clusters in RNA-seq and ATAC-seq data, one enriched for inflammatory pathways (e.g., *IL15, SIGLEC1*) and the other for cell-death pathways (e.g., *BCL2A1, GADD45B*), suggests distinct microglial activation states in HIV. These states may reflect a balance between pro-inflammatory and pro-apoptotic responses, with implications for neuroinflammation and neuronal survival. Exclusive dysregulation of certain pathways in glial cells provided further insights, particularly in microglia and astrocytes, where IL-17 and IL-1β production were notably upregulated, signaling heightened inflammation. We also found toll-like receptor pathway exclusively upregulated in microglia, its role in inflammation and microglial HIV latency is well studied^152,186,187^. Additionally, pathways related to xenobiotic metabolism, viral carcinogenesis, T-cell receptor signaling and ephrin signaling^153^ were also exclusively dysregulated in microglia, emphasizing their specialized immune function in the central nervous system. Of particular interest was the cell-type specific perturbation of the *NRF2* pathway, which was upregulated in microglia but downregulated in OPCs, see Figure-8 (d). Given that *NRF2* activation is known to inhibit HIV replication in macrophages, but not in T-cells, the opposing regulation in these two glial cell types highlights the differential impact of HIV on cellular immune responses in the CNS. This finding is further supported by the unique role microglia, as CNS-resident macrophages, play in immune surveillance and neuroinflammation^188,189^.

Overall, the enriched pathways in glial cells showed a pattern of alterations that align with changes seen in other cell types, particularly those associated with mitochondrial dysfunction, inflammation, and reduced protein synthesis. Additionally, upregulated pathways related to extracellular matrix interactions and integrin-mediated signaling point to changes in cell adhesion and communication, while hematopoietic cell lineage and pattern recognition receptor signaling further highlight immune activation^147,148,190^. We also observed downregulation of pathways involved in memory, serotonin neurological impact of HIV, particularly given the documented sleep disturbances in people living with HIV and their association with neurodegenerative diseases like AD^191,192^. Other glial-specific perturbations included monocyte chemotaxis regulation, which is linked to increased HIV infectivity, and vacuolar lumen dysregulation, suggesting compromised cellular integrity and increased susceptibility to infections.

The ATAC-seq analysis, performed on 85,234 cells from 12 samples, revealed DARs that provide insights into the epigenetic regulation of DEGs. The significant overlap between DEGs and DARs, particularly in neurons, Figure-8 (h), underscores the role of chromatin accessibility in modulating gene expression in neuroHIV. We found DEGs that contain DARs to be most prominent in the excitatory and inhibitory neuronal cell classes, other studies^184,193^ suggest that most people with HIV, are likely to experience HIV associated dementia or cognitive decline, lending support to our finding that neuronal cells present the maximum differential regulation among genes and chromatin accessibility sites. We found dysregulation of multiple collagen genes in neurons, see Figure-10 (*COL11A1, COL12A1, COL13A1, COL22A1, COL9A2* and *COL9A3*) and multiple pathways related to extra-cellular matrix; collagen genes’ expression in neurons is documented^194,195^ and collagen deposition has been found to deplete and disrupt homeostasis of T-cells in HIV^196,197^ and has been investigated as a therapeutic avenue for repair and regeneration of central nervous system neurons in neurodegenerative diseases and aging^198^. We also found dysregulation of multiple voltage gated potassium channel genes like *KCNG2, KCNQ4* and *KCNS3* in neurons, immune cells and vascular cells and voltage gated potassium channel disintegration has been implicated in neurocognitive decline in HIV^199^. We also found neuronal dysregulation of phosphatase genes *PTPRT* and *PTPRU*, which have been implicated in neurodevelopmental diseases^200^. Also in neuronal cells, we found T-box transcription factors, *TBX15* and *TBX19* with positive chromatin accessibility and differential expression, a paralog *TBX21* (or T-bet) has been studied for its association with accumulation of B-cells in HIV infection in lymph nodes^201^. Lastly, we found neuronal DEGs with DARs to be significantly enriched in chemical synaptic transmission and dysregulated components in both pre and post synaptic membranes, see Figure-11, suggesting a critical role of chromatin accessibility changes in synaptodendritic dysregulations in HIV. The enrichment of neuronal DEGs with DARs in synaptic processes, as identified using the SynGO database, suggests that HIV-induced epigenetic changes directly impact synaptic function. Dysregulation of chemical synaptic transmission and pre- and postsynaptic membrane components may contribute to the synaptic loss and cognitive impairments characteristic of neurocognitive decline associated with HIV. These findings align with previous studies linking synaptic dysfunction to neurodegeneration, reinforcing the hypothesis that HIV and AD share molecular pathways affecting synaptic integrity.

A limitation of the study is the relatively low number of samples available for the present analysis, which was suboptimal for investigating stages of neurological decline in PWH and combined analysis of epigenome.

This study reveals a complex landscape of cell-type-specific transcriptional and epigenetic changes in the pre-frontal cortex of virally suppressed PWH, highlighting parallels with AD and other neurodegenerative diseases. The predominance of downregulated DEGs, upregulation of neuroinflammatory markers, and dysregulation of metabolic and DNA repair pathways underscore the multifaceted impact of HIV on the central nervous system. The integration of snRNA-seq and snATAC-seq data provides a robust framework for understanding the molecular underpinnings of neurological impact associated with HIV and suggests potential therapeutic avenues to address neuroinflammation and neuronal dysfunction in HIV.

## Supplementary Material

Supplementary Files

This is a list of supplementary files associated with this preprint. Click to download.

- SupplementaryFigure3.pdf
- SupplementaryFigure1.pdf
- SupplementaryFigure2.pdf
- SupplementaryTable1.xlsx
- SupplementaryTable6.xlsx
- SupplementaryTable2.xlsx
- SupplementaryTable4.xlsx
- SupplementaryTable3.xlsx
- SupplementaryTable5.xlsx

## Data Availability

We analyzed samples from the SCORCH (Single Cell Opioid Responses in the Context of HIV) consortium^13^. The data in this study are publicly accessible at NEMO Archive (RRID:SCR 002001) under identifier nemo: x. Code to analyze the data is on a private GitHub repository and can be requested from the corresponding author under controlled use conditions due to human privacy regulations.
